# Reproductive history and progressive multiple sclerosis risk in women

**DOI:** 10.1093/braincomms/fcaa185

**Published:** 2020-11-17

**Authors:** Burcu Zeydan, Elizabeth J Atkinson, Delana M Weis, Carin Y Smith, Liliana Gazzuola Rocca, Walter A Rocca, Brian Mark Keegan, Brian G Weinshenker, Kejal Kantarci, Orhun H Kantarci

**Affiliations:** 1 Department of Neurology, Mayo Clinic, Rochester, MN 55905, USA; 2 Department of Radiology, Mayo Clinic, Rochester, MN 55905, USA; 3 Center for Multiple Sclerosis and Autoimmune Neurology, Mayo Clinic, Rochester, MN 55905, USA; 4 Women’s Health Research Center, Mayo Clinic, Rochester, MN 55905, USA; 5 Division of Biomedical Statistics and Informatics, Department of Health Sciences Research, Mayo Clinic, Rochester, MN 55905, USA; 6 Division of Epidemiology, Department of Health Sciences Research, Mayo Clinic, Rochester, MN 55905, USA

**Keywords:** progressive multiple sclerosis, menarche, pregnancy, menopause, oestrogen

## Abstract

Being a woman is one of the strongest risk factors for multiple sclerosis. The natural reproductive period from menarche to natural menopause corresponds to the active inflammatory disease period in multiple sclerosis. The fifth decade marks both the peri-menopausal transition in the reproductive aging and a transition from the relapsing-remitting to the progressive phase in multiple sclerosis. A short reproductive period with premature/early menopause and/or low number of pregnancies may be associated with an earlier onset of the progressive multiple sclerosis phase. A cross-sectional study of survey-based reproductive history in a multiple sclerosis clinical series enriched for patients with progressive disease, and a case–control study of multiple sclerosis and age/sex matched controls from a population-based cohort were conducted. Menarche age, number of complete/incomplete pregnancies, menopause type and menopause age were compared between 137 cases and 396 control females. Onset of relapsing-remitting phase of multiple sclerosis, progressive disease onset and reaching severe disability (expanded disability status scale 6) were studied as multiple sclerosis-related outcomes (*n* = 233). Menarche age was similar between multiple sclerosis and control females (*P* = 0.306). Females with multiple sclerosis had fewer full-term pregnancies than the controls (*P* < 0.001). Non-natural menopause was more common in multiple sclerosis (40.7%) than in controls (30.1%) (*P* = 0.030). Age at natural menopause was similar between multiple sclerosis (median, interquartile range: 50 years, 48–52) and controls (median, interquartile range: 51 years, 49–53) (*P* = 0.476). Nulliparous females had earlier age at progressive multiple sclerosis onset (mean ± standard deviation: 41.9 ± 12.5 years) than females with ≥1 full-term pregnancies (mean ± standard deviation: 47.1 ± 9.7 years) (*P* = 0.069) with a pregnancy-dose effect [para 0 (mean ± standard deviation: 41.9 ± 12.5 years), para 1–3 (mean ± standard deviation: 46.4 ± 9.2 years), para ≥4 (mean ± standard deviation: 52.6 ± 12.9 years) (*P* = 0.005)]. Menopause age was associated with progressive multiple sclerosis onset age (*R*^2^ = 0.359, *P* < 0.001). Duration from onset of relapses to onset of progressive multiple sclerosis was shorter for females with premature/early menopause (*n* = 26; mean ± standard deviation: 12.9 ± 9.0 years) than for females with normal menopause age (*n* = 39; mean ± standard deviation: 17.8 ± 10.3 years) but was longer than for males (mean  ±standard deviation: 10.0 ± 9.4 years) (*P* = 0.005). There was a pregnancy-dose effect of age at expanded disability status scale 6 (para 0: 43.0 ± 13.2 years, para 1–3: 51.7 ± 11.3 years, para ≥4: 53.5 ± 4.9 years) (*P* = 0.013). Age at menopause was associated with age at expanded disability status scale 6 (*R*^2^ = 0.229, *P* < 0.003). Premature/early menopause or nulliparity was associated with earlier onset of progressive multiple sclerosis with a ‘dose effect’ of pregnancies on delaying progressive multiple sclerosis and severe disability. Although causality remains uncertain, our results suggest a beneficial impact of oestrogen in delaying progressive multiple sclerosis. If confirmed in prospective studies, our findings have implications for counselling women with multiple sclerosis about pregnancy, surgical menopause and menopausal hormone therapy.

## Teaser

Premature/early menopause or nulliparity was associated with earlier progressive multiple sclerosis onset with a ‘dose effect’ of pregnancies on delaying progressive multiple sclerosis and severe disability. Zeydan *et al.*’s findings suggest a beneficial impact of oestrogen in delaying progressive multiple sclerosis and may have implications for counselling women on reproductive health.

## Graphical Abstract

**Graphical Abstract:**
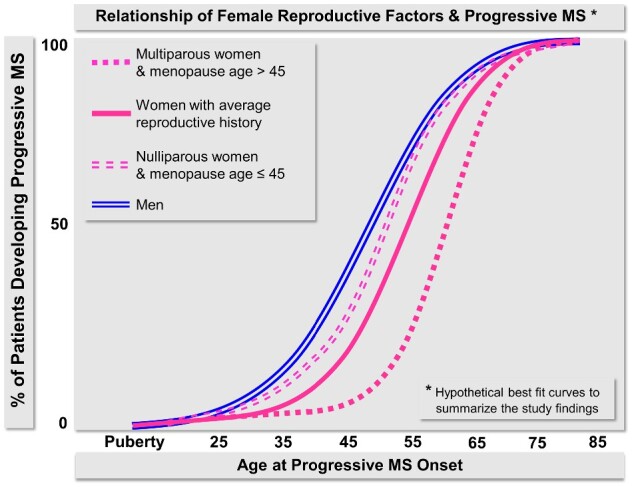

## Introduction

A complex interaction between genetic, epigenetic, hormonal and environmental factors is likely responsible for the risk of developing multiple sclerosis and also for the variability of disease course in multiple sclerosis (Kantarci *et al.*, 2002; Kantarci, 2016). One of the strongest risk factors for developing multiple sclerosis is being a woman. Hormonal maturation influences sex-specific multiple sclerosis risk: although the frequency of multiple sclerosis is similar among girls and boys before puberty, it dramatically increases in girls after puberty (Ghezzi, 2005). In some populations, the post-pubertal female predominance appears to have increased over time with a current female to male ratio of 3:1, higher than previous estimates of 2:1 (Murray *et al.*, 2004; Koch-Henriksen and Sorensen, 2010; Trojano *et al.*, 2012). This increasing time trend suggests that, beyond genetics, there are both hormonal and environmental contributions to the sex differences in multiple sclerosis (Kantarci, 2011).

Following symptom onset, sex-specific factors continue to influence disease course in multiple sclerosis. The relapsing-remitting phase starts at a younger age in women than men, women have more frequent relapses then men, and men enter the progressive phase earlier then women (Confavreux *et al.*, 1980; Weinshenker *et al.*, 1989*a*, 1991; Runmarker and Andersen, 1993; Kantarci *et al.*, 1998). Men accumulate disability faster and reach severe disability milestones earlier in the relapsing-remitting phase; whereas, after the onset of the progressive phase, women accumulate disability faster and catch up to similar levels of severe disability as men (Paz Soldan *et al.*, 2015). In patients with multiple sclerosis onset after age 50, women and men have similar disability worsening rates (Bove *et al.*, 2012). The fifth decade critically marks the transition from the relapsing-remitting phase to the progressive phase in multiple sclerosis in both sexes (Confavreux and Vukusic, 2006; Koch *et al.*, 2007; Tutuncu *et al.*, 2013). Interestingly, the fifth decade also marks the peri-menopausal transition in the reproductive aging of most women.

The natural reproductive period from menarche to natural menopause, interspaced with pregnancy(s) and puerperium, corresponds to the most active inflammatory disease period in women with multiple sclerosis. Relapse frequency decreases especially in the third trimester of pregnancy; however, within 3 months postpartum, the relapse frequency reverts to pre-pregnancy levels (Confavreux *et al.*, 1998). Therefore, the interaction between sex hormones and the immune system emerges as a potentially important factor in determining relapse frequency in women with multiple sclerosis. Oestradiol contributes to neuroprotection by promoting neuronal plasticity and remyelination, and by reducing astrogliosis (Azcoitia *et al.*, 2011). Through regulatory roles on astrocyte and microglia function, oestradiol also reduces excitotoxicity, reduces expression of pro-inflammatory molecules and protects mitochondrial function. These changes ultimately lead to reduced CNS inflammation and increased neuronal survival, which are biological mechanisms related to neurodegeneration and to the progressive phase of multiple sclerosis (Arevalo *et al.*, 2010; Acaz-Fonseca *et al.*, 2014).

We evaluated the effect of reproductive history on the development of progressive multiple sclerosis in women. Given the associations described, we hypothesized that a short reproductive period with premature/early menopause and/or low number of pregnancies would be associated with an earlier onset of the progressive phase of multiple sclerosis in women.

## Materials and methods

### Study type

Our study had two components: (i) a *cross-sectional study* of survey-based reproductive history in multiple sclerosis and (ii) a *case–control study* of menarche, pregnancy and menopause in multiple sclerosis and matched controls.

### Study population

**Figure 1:**
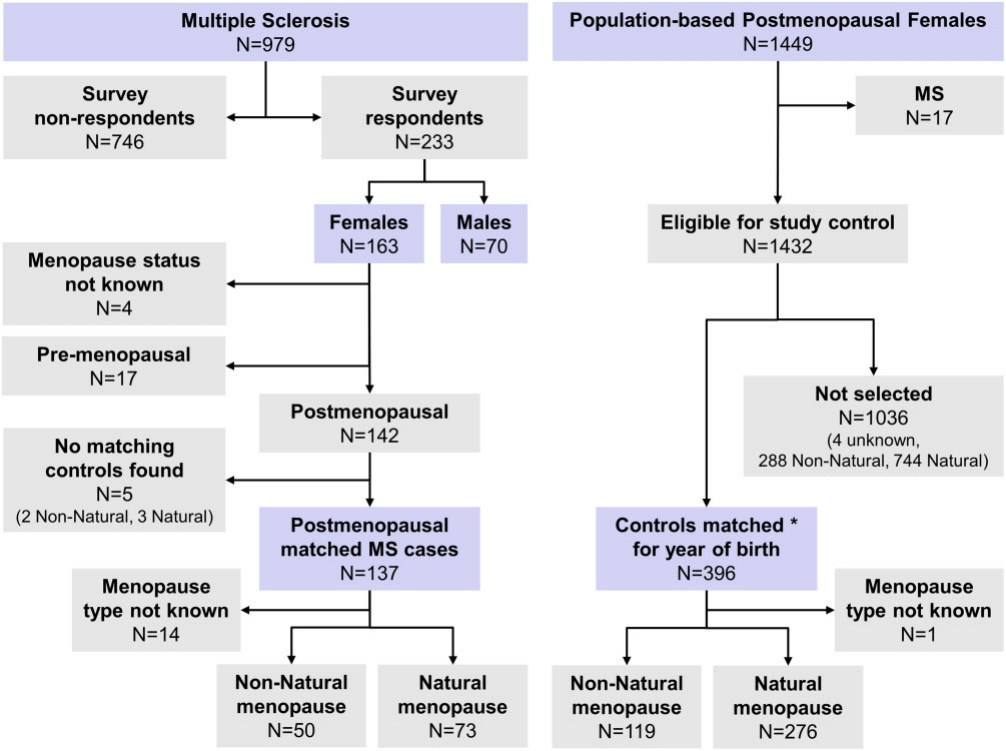
**Study sample.** Participants from the multiple sclerosis clinical series and the Mayo Clinic Cohort Study of Oophorectomy and Aging-2. The multiple sclerosis clinical series was enriched for progressive multiple sclerosis. Of the 979 patients, 233 responded to the survey (163 females, 70 males). Of the 163 respondent females with multiple sclerosis, 142 were postmenopausal. The control females from the general population were derived from the Mayo Clinic Cohort Study of Oophorectomy and Aging-2. After excluding patients with multiple sclerosis (*n* = 17), postmenopausal control females (*n* = 396) were randomly 3:1 matched for year of birth to postmenopausal females from the multiple sclerosis study cohort. *A 3:1 matching was available for 128 cases, 2:1 was available for three cases, 1:1 was available for six cases and no controls were available for five cases. By excluding the five multiple sclerosis patients without matching controls, case–control analyses were conducted in 137 postmenopausal females with multiple sclerosis and 396 year of birth matched control females

Details of our larger multiple sclerosis clinical series enriched for patients with progressive disease were previously published (Fig. 1) (Tutuncu *et al.*, 2013; Paz Soldan *et al.*, 2015). A survey to collect information on the impact of environmental factors and reproductive history was sent to 979 participants with a 24% response rate (*n* = 233; 163 females, 70 males). Among 163 respondent females, 142 were postmenopausal.

The Mayo Clinic Cohort Study of Oophorectomy and Aging-2 is a population-based cohort study (1988–2007) that includes referent females who are representative of the general female population of Olmsted County, Minnesota (Rocca *et al.*, 2017). From this study cohort, we identified a subset of 1449 postmenopausal females who were born between 1935 and 1975 (menopause age range: 11–61 years) and who had undergone surgical or non-surgical menopause. From this subset of 1449 individuals, 17 females who had a multiple sclerosis diagnosis were excluded. Of the remaining 1432 females, individuals were randomly matched to 142 postmenopausal females with multiple sclerosis for year of birth (restricted to ±3 years), with an attempted 3:1 ratio. The 3:1 ratio was available for 128 cases, a 2:1 ratio was available for three cases and a 1:1 was available for six cases. No controls were available for five cases. By excluding the five patients with multiple sclerosis without matching controls, case–control analyses were conducted in 137 postmenopausal females with multiple sclerosis and 396 matched control females.

### Study consent

The study protocol was approved by the Mayo Clinic and Olmsted Medical Center Institutional Review Boards. Informed consent was obtained from each participant with multiple sclerosis. The data collection for the control females was historical, and these females provided a general consent to use their medical records for research (Minnesota legal requirement).

### Data collection

The extensive multiple sclerosis survey questionnaire included environmental, lifestyle, personal and hereditary medical history questions. Participants were given the option to complete surveys electronically or via a phone interview by a trained study coordinator in case of electronic access difficulties. After a pre-specified number of contact attempts, if no response was received, patients were considered non-responders. For responders, data were cross-checked against Mayo Clinic medical records to obtain additional details or when clarification was needed. For this study, we extracted the specific answers to questions on reproductive history related to menarche age, number of complete and incomplete pregnancies, menopause type and menopause age.

All reproductive history data in the control group were originally abstracted from all of the medical records included in the medical records linkage system of the Rochester Epidemiology Project as described previously (Rocca *et al.*, 2017).

### Definition of reproductive history variables

*Menarche age* as recorded in the charts and/or recalled by the patient was treated as a continuous variable.

*Pregnancy* was studied in two ways: (i) *full-term pregnancies only (para)*, which was further classified into three groups as nulliparous (para 0), para 1–3 and para ≥4; (ii) *any pregnancy regardless of the outcome (gravida)* (to investigate if the pregnancies ending in abortion and miscarriage made a difference in our findings) also classified into three groups as gravida 0, gravida 1–3 and gravida ≥4.

*Menopause type* was classified as natural (due to natural physiology) and non-natural (females who went into menopause due to surgical intervention or chemo/radiotherapy). In the control group, *menopause status* was classified as pre-menopausal (females who are still cycling) and postmenopausal (females who have no menses in the prior 12 months or no menses due to surgery or chemo/radiotherapy and age at that year was treated as the *age of menopause*). Within the survey, patient’s recollection of the *age of menopause* was used. Depending on the study question, *age of menopause* was either treated as a continuous variable or classified by commonly accepted cut-off as premature-early (age ≤45 years) or normal (age >45 years) (Shifren *et al.*, 2014).

### Definition of outcome variables

We studied three multiple sclerosis-related outcomes: (i) onset of relapsing-remitting phase of multiple sclerosis; (ii) developing a progressive disease course; and (iii) reaching severe disability.

Onset of relapsing-remitting phase was defined as the first clinical attack (clinically isolated syndrome) in patients with relapsing-remitting multiple sclerosis, single-attack progressive multiple sclerosis characterized by a single clinical attack preceding progressive multiple sclerosis and secondary progressive multiple sclerosis (SPMS) characterized by multiple attacks preceding progressive multiple sclerosis. For practical purposes, single-attack progressive multiple sclerosis and SPMS were collapsed into SPMS. As patients with primary progressive multiple sclerosis (52 of 163 respondent females) do not have a clinically documented relapsing-remitting phase preceding the progressive disease, they were excluded from this specific outcome analysis.

Progressive disease phase was defined as an insidious and irreversible worsening of neurological symptom(s) due to multiple sclerosis lasting for ≥1 year (Lublin and Reingold, 1996; Lublin *et al.*, 2014). Reaching severe disability was defined by needing unilateral assistance to walk corresponding to the ordinal value of expanded disability status scale of 6 (EDSS 6) (Kurtzke, 1983). For these outcomes, we included primary progressive multiple sclerosis patients in the analyses together with SPMS. Age at progressive multiple sclerosis onset, age at EDSS 6, time from onset of relapsing-remitting phase to onset of progressive multiple sclerosis or time from onset of relapsing-remitting phase to reaching EDSS 6 were studied.

### Statistics and data analyses

We initially analysed any potential selection bias in patients who responded to the survey by comparing sex distribution, age at progressive multiple sclerosis onset, percentage of patients who developed progressive multiple sclerosis and percentage of patients who had reached EDSS 6 using Kruskal–Wallis rank sum test or Pearson’s chi-squared test as appropriate.

Next, we studied multiple sclerosis-specific changes in reproductive history by comparing menarche age, pregnancy numbers, menopause type and menopause age between patients and matched controls using Kruskal–Wallis rank sum test or Pearson’s chi-squared test as appropriate.

Finally, we studied the association between menarche age, pregnancy variables, menopause type and menopause age with age at progressive multiple sclerosis onset both univariately and in multivariable analyses using Kruskal–Wallis rank sum test, Pearson’s chi-squared test, linear model ANOVA or linear regression models as appropriate. We then repeated the analyses for the disability outcome.

### Data availability

Anonymized data may be shared upon request from a qualified academic investigator for the only purpose of replicating procedures and results presented in this article. Data sharing will follow our established institutional policies.

## Results

### Demographic and clinical characteristics of patients with multiple sclerosis

Comparing demographics of the survey respondents with the non-respondents: Female to male ratio was 2.2 versus 1.6 (*P* = 0.073), relapsing-remitting multiple sclerosis frequency was 14% versus 8% (*P* = 0.024), SPMS frequency was 47.4% versus 57.8% (*P* = 0.009), primary progressive multiple sclerosis frequency was 38.6% versus 33.3% (*P* = 0.134), age at progressive multiple sclerosis onset was 46.7 ± 10.6 versus 45.0 ± 10.0 (*P* = 0.017) and frequency of patients who reached EDSS 6 or higher was 83.3% versus 64.8% (*P* < 0.001). Respondent females had progressive multiple sclerosis onset (mean ± standard deviation [SD]: 45.7 ± 10.5 years) at the expected age distribution from the original cohort of 983 patients (mean ± SD: 45.5 ± 10.0 years) (Tutuncu *et al.*, 2013). However, males had a slightly older age at progressive multiple sclerosis onset (mean ± SD: 48.7 ± 10.4 years) than females (*P* = 0.059). Females expectedly had an earlier relapsing-remitting phase onset (mean ± SD: 36.3 ± 11.6 years) than males (mean ± SD: 42.6 ± 12.4 years) (*P* < 0.001).

**Table 1:** Participants’ characteristics

	Control Females	Multiple sclerosis		*P*
		Females	Males	
All survey respondents (*N*)		163	70	
Age at relapsing-remitting multiple sclerosis onset (median; IQR)		35.4 (26.4–45.8)	42.3 (32.8–52.9)	<0.001
Age at progressive multiple sclerosis onset (median; IQR)		47.4 (39.7–51.9)	49.9 (40.5–55.3)	0.059
Time from relapsing-remitting multiple sclerosis to progressive multiple sclerosis onset (median years; IQR)		4 (0–16)	0 (0–5.8)	0.016
Age at survey (median; IQR)		62.6 (54.2–60.7)	66.3 (56.9–70.9)	0.043
Progressive multiple sclerosis at the time of survey (*N*; %)		131 (80.4%)	65 (92.9%)	0.017
EDSS at last follow-up (median; IQR)		6 (3.5–6.5)	6 (4.1–6.4)	0.731
Postmenopausal matched sets (*N*)^*^	396	137		
Age at menarche (median; IQR)	12 (12–13)	13 (12–13.2)		0.306
All pregnancies (median; IQR)	3 (2–4)	2 (1–3)		0.001
Full-term pregnancies (median; IQR)	2 (2–3)	2 (1–3)		<0.001
Non-natural menopause (*N*; %)	119 (30.1%)	50 (40.7%)		0.030
Age at menopause (median; IQR)	50 (43–52)	49 (44.5–52)		0.791
Age at non-natural menopause (median; IQR)	39 (34–46)	44.5 (38–48)		0.009
Age at natural menopause (median; IQR)	51 (49–53)	50 (48–52)		0.476

* Age at menarche was missing for 78 controls and 13 multiple sclerosis females, number of pregnancies was missing for two controls and six multiple sclerosis females, number of full-term pregnancies was missing for two controls and four multiple sclerosis females, type of menopause was missing for one control and 14 multiple sclerosis females, age at menopause was missing for 10 controls and nine multiple sclerosis females, age at non-natural menopause was missing for 0 controls and 0 multiple sclerosis females and age at natural menopause was missing for nine controls with natural menopause and four multiple sclerosis females with natural menopause. IQR: interquartile range; EDSS: expanded disability status scale.

At the time of the survey, males with multiple sclerosis (*n* = 70, median age, interquartile range [IQR]: 66.3 years, 56.9–70.9) were slightly older than females with multiple sclerosis (*n* = 163, median age, IQR: 62.6 years, 54.2–60.7) (*P* = 0.043) (Table 1). Given the older age group of the study respondents, most patients had already entered the progressive phase of multiple sclerosis. However, males (92.9%) were still more likely to be in the progressive phase than females (80.4%) (*P* = 0.017). Similarly, with most patients already in the progressive phase and being older than 50 years of age, they had a median EDSS of 6 with no difference between females and males (*P* = 0.731).

### Reproductive history characteristics of females with multiple sclerosis compared to control females

**Figure 2:**
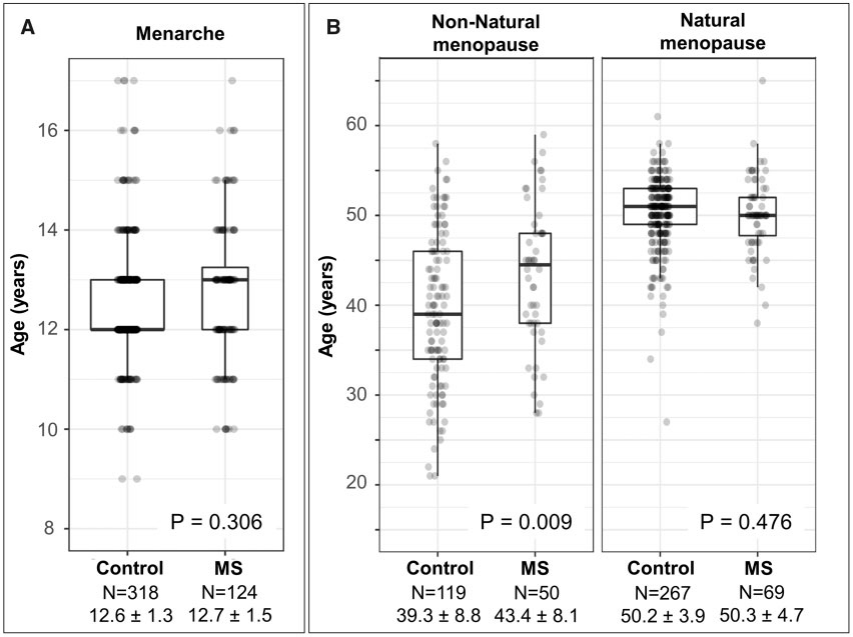
**(**A**) Age at menarche in multiple sclerosis and controls.** Menarche age did not differ between females with multiple sclerosis and control females. (**B**) Age at menopause in multiple sclerosis and controls. Age at natural menopause did not differ between females with multiple sclerosis and control females. Age at non-natural menopause was older in multiple sclerosis than control females. Type of menopause was not available in 14 females with multiple sclerosis and one control woman as shown in Fig. 1

Menarche age in females with multiple sclerosis was not different from control females (*P* = 0.306) (Table 1 and Fig. 2A).

Females with multiple sclerosis had lower number of pregnancies than the control females (*P* = 0.001) and lower number of full-term pregnancies than the control females (*P* < 0.001) (Table 1). Nulliparity was slightly more common in females with multiple sclerosis (21%) than in control females (14%) (*P* = 0.062).

Overall age at menopause was similar between females with multiple sclerosis (median, IQR: 49 years, 44.5–52) and control females (median, IQR: 50 years, 43–52) (*P* = 0.791) (Table 1). As seen in Fig. 2B, age at non-natural menopause was expectedly younger overall than age at natural menopause regardless of having had multiple sclerosis or not. However, while age of natural menopause was indistinguishable in females with multiple sclerosis (median, IQR: 50 years, 48–52) and control females (median, IQR: 51 years, 49-53) (*P* = 0.476), at the onset of non-natural menopause, females with multiple sclerosis were older (median, IQR: 44.5 years, 38–48) than control females (median, IQR: 39 years, 34–46) (*P* = 0.009) (Fig. 2B). Additionally, excluding patients (*n* = 14) and controls (*n* = 1) without a known menopause type, non-natural menopause was more commonly observed in females with multiple sclerosis (40.7%) than control females (30.1%) (*P* = 0.030) (Table 1).

### Reproductive history characteristics of females with MS and relapsing-remitting phase onset

#### Menarche and relapsing-remitting phase onset

Of the females with SPMS who had a relapsing-remitting phase preceding progressive multiple sclerosis, 84% were known to have had menarche before relapsing-remitting phase onset. In this sub-group, age at menarche was not associated with age at onset of relapsing-remitting phase (*R*^2^ = 0.012, *P* = 0.590), number of pregnancies (*R*^2^ = 0.029, *P* = 0.406), number of full-term pregnancies (*R*^2^ = 0.054, *P* = 0.251) or nulliparity (*R*^2^ = 0.000, *P* = 0.949).

#### Pregnancy and relapsing-remitting phase onset

Nulliparous group (mean ± SD: 27.5 ± 7.0 years) had an earlier age at onset of relapsing-remitting phase than females with ≥1 parity (mean ± SD: 33.0 ± 9.4 years) (*P* = 0.015). There was a dose-dependent association between parity and older age at relapsing-remitting phase onset (para ≥4: 35.8 ± 9.8 years, para 1–3: 32.4 ± 9.3 years, para 0: 27.5 ± 7.0 years) (*P* = 0.012).

Using gravida instead of parity, differences were similar to the findings for parity: nulligravid group (mean ± SD: 28.2 ± 7.1 years) versus gravida 1–3 (mean ± SD: 31.7 ± 9.4 years) versus gravida ≥4 (mean ± SD: 35.0 ± 9.4 years) (*P* = 0.058).

In the absence of data on age at pregnancies from the survey, we could not specifically study the directionality of the association between pregnancy number and age at relapsing-remitting phase onset.

#### Menopause and relapsing-remitting phase onset

Of the females with SPMS who had a relapsing-remitting phase preceding progressive multiple sclerosis, 69% were known to have had menopause after relapsing-remitting phase onset. In this sub-group, age at menopause was not associated with age at onset of relapsing-remitting phase (*R*^2^ = 0.156, *P* = 0.069), number of pregnancies (*R*^2^ = 0.037, *P* = 0.389), number of full-term pregnancies (*R*^2^ = 0.032, *P* = 0.425), nulliparity (*R*^2^ = 0.038, *P* = 0.383) or natural versus non-natural menopause (*R*^2^ = 0.040, *P* = 0.397). When the analysis was restricted to females with only natural menopause or only non-natural menopause, the results were similar (data not shown).

### Reproductive history characteristics of females with multiple sclerosis and progressive multiple sclerosis onset

#### Menarche and progressive multiple sclerosis onset

Age at menarche was not associated with age at progressive multiple sclerosis onset (*R*^2^ = 0.008, *P* = 0.337), age at relapsing-remitting phase onset (*R*^2^ = 0.001, *P* = 0.749), number of pregnancies (*R*^2^ = 0.009, *P* = 0.328), number of full-term pregnancies (*R*^2^ = 0.005, *P* = 0.452) or nulliparity (*R*^2^ = 0.000, *P* = 0.869).

#### Pregnancy and progressive multiple sclerosis onset

**Figure 3:**
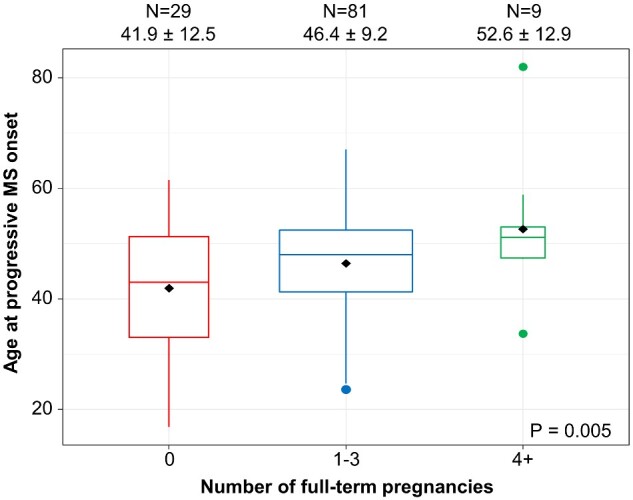
**The association between number of pregnancies and age at progressive multiple sclerosis onset.** Higher number of pregnancies was associated with older age at progressive multiple sclerosis onset (mean ± SD) with a dose-dependent effect: nulliparous females (para 0) had the youngest age at progressive multiple sclerosis onset, females with para 1–3 had an intermediate age at progressive multiple sclerosis onset and females with para ≥4 had the oldest age at progressive multiple sclerosis onset

Nulliparous females had earlier age at progressive multiple sclerosis onset (mean ± SD: 41.9 ± 12.5 years) compared to females with ≥1 full-term pregnancies (mean ± SD: 47.1 ± 9.7 years) (*P* = 0.069), an effect that became more evident with a dose-dependent association between parity and age at progressive multiple sclerosis onset (Fig. 3); females with para ≥4 (mean ± SD: 52.6 ± 12.9 years), para 1–3 (mean ± SD: 46.4 ± 9.2 years) and para 0 (mean ± SD: 41.9 ± 12.5 years) (*P* = 0.005).

To study if the absence of a relapsing-remitting phase before developing progressive multiple sclerosis (primary progressive multiple sclerosis) impacted our findings, we also restricted the analyses to the SPMS-only sub-group (*n* = 76). Nulliparous females again had earlier age at progressive multiple sclerosis onset (mean ± SD: 41.5 ± 9.2 years) than females with ≥1 parity (mean ± SD: 47.3 ± 10.6 years) (*P* = 0.039). There was again a dose-dependent association between parity and age at progressive multiple sclerosis onset (para ≥4: 52.6 ± 12.9 years, para 1–3: 46.2 ± 9.9 years, para 0: 41.5 ± 9.2 years) (*P* = 0.010).

In the absence of data on age at pregnancies from the survey, the analyses were repeated by further excluding females who were known to be pre-menopausal at the time of progressive multiple sclerosis onset, therefore essentially eliminating any chance of further pregnancies. The same directionality of dose effect of pregnancy numbers on progressive multiple sclerosis onset remained (para ≥4: 58.5 ± 13.5 years, para 1–3: 52.6 ± 8.5 years, para 0: 48.0 ± 8.4 years) (*P* = 0.060). When the analysis was repeated using gravida, we observed similar dose effect of pregnancies on progressive multiple sclerosis onset (gravida ≥4: 58.5 ± 12.1 years, gravida 1–3: 52.2 ± 8.4 years, gravida 0: 47.5 ± 9.0 years) (*P* = 0.049).

#### Menopause and progressive multiple sclerosis onset

**Figure 4:**
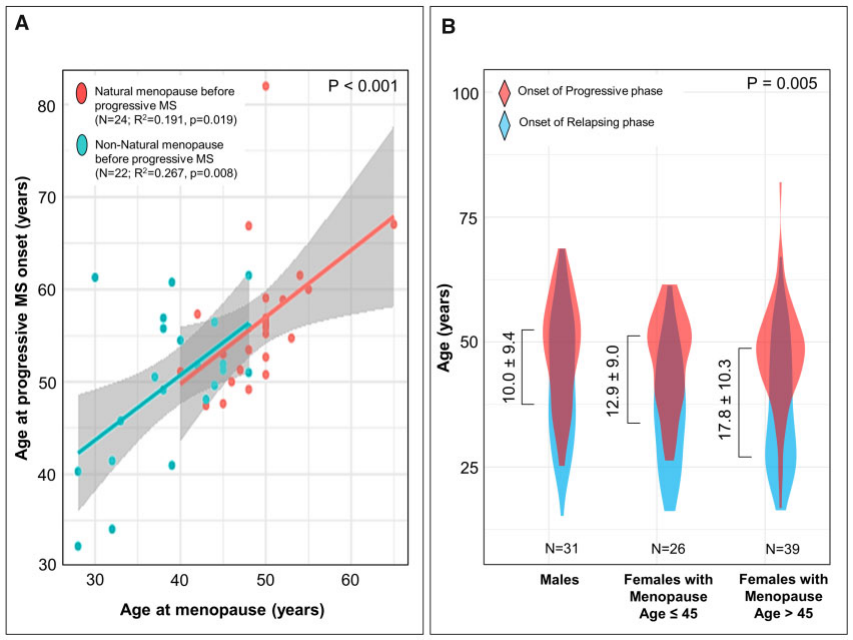
**(**A**) Age at progressive multiple sclerosis onset in females who had menopause before progressive multiple sclerosis onset.** Age at menopause was associated with age at progressive multiple sclerosis onset in the entire group (*R*^2^ = 0.359, *P* < 0.001) as well as in females with natural menopause and females with non-natural menopause. (**B**) Duration from onset of relapsing-remitting phase to onset of progressive multiple sclerosis in SPMS patients. Relapsing-remitting (pre-progression) disease duration (mean ± SD) was compared in three groups (*P* = 0.005): males, females with premature/early menopause (menopause age ≤45 years) and females with normal age at menopause (menopause age >45 years). Relapsing-remitting disease duration was shortest in males, intermediate in females with premature/early menopause and longest in females with normal age at menopause

Of the females who had progressive multiple sclerosis (SPMS and primary progressive multiple sclerosis), 36% were known to have had menopause before progressive multiple sclerosis onset. In this sub-group, age at menopause was associated with age at progressive multiple sclerosis onset (*R*^2^ = 0.359, *P* < 0.001) (Fig. 4A), whether or not the analysis was restricted to natural (*n* = 24; *R*^2^ = 0.191, *P* = 0.019) or non-natural (*n* = 22; *R*^2^ = 0.267, *P* = 0.008) menopause sub-groups (Fig. 4A).

In the SPMS sub-group, duration from onset of relapses to onset of progressive multiple sclerosis in females with premature/early menopause (*n* = 26; mean ± SD: 12.9 ± 9.0 years) was shorter than in females with normal age at menopause (*n* = 39; mean ± SD: 17.8 ± 10.3 years) and was longer than males (mean ± SD: 10.0 ± 9.4 years) (*P* = 0.005) (Fig. 4B). When the analysis was restricted to patients with SPMS onset after menopause, duration from onset of relapses to onset of progressive multiple sclerosis was again shorter in females with premature/early menopause (*n* = 17; mean ± SD: 13.9 ± 8.8 years) than in females with normal age at menopause (*n* = 10; mean ± SD: 25.4 ± 13.3 years) (*P* = 0.018).

#### Multivariable analyses of reproductive history milestones and progressive multiple sclerosis onset

For this analysis, females who were pre-menopausal at the time of survey were excluded (to eliminate any chance of any further pregnancy). In the remaining 112 females and 65 males, we fit univariate and multivariable models with the following variables known as of menopause onset: male sex, being a pre-menopausal woman at the time of progressive multiple sclerosis onset, menarche age, parity status (≥1 versus 0), gravida status (≥1 versus 0), menopause type (non-natural versus natural), menopause age, age at relapsing-remitting phase onset and previous use of disease-modifying therapy (longer than 3 months and before progressive multiple sclerosis onset).

Although male sex (*P* = 0.023) or older age at relapsing-remitting phase onset (*P* < 0.001) emerged as additional independent predictors of the age at progressive multiple sclerosis onset, disease-modifying therapy use prior to progressive multiple sclerosis onset did not have an effect on age at progressive multiple sclerosis onset (*P* = 0.099).

### Reproductive history characteristics of females with multiple sclerosis and reaching EDSS 6

Of the respondent females with multiple sclerosis, 73% were known to have reached the severe disability milestone of EDSS 6 at the time of the survey at a mean age of 49.3 ± 12.0 years.

Age at menarche was not associated with reaching EDSS 6 (*P* = 0.912). There was a dose-dependent association between parity and age at EDSS 6 (para ≥4: 53.5 ± 4.9 years, para 1–3: 51.7 ± 11.3 years, para 0: 43.0 ± 13.2 years) (*P* = 0.013). There was also a dose-dependent association between gravida and age at EDSS 6 (gravida ≥4: 53.3 ± 9.0 years, gravida 1–3: 50.6 ± 11.6 years, gravida 0: 41.6 ± 13.8 years) (*P* = 0.015). After eliminating females who reached EDSS 6 before menopause and who still had pregnancy potential, the sample size (*n* = 17) was too small to complete meaningful statistical analyses (data not shown).

Of the females who had reached EDSS 6, 52% were known to have had menopause before the EDSS 6 milestone. In this sub-group, age at menopause was associated with age at EDSS 6 (*R*^2^ = 0.229, *P* < 0.003). Similar associations were observed when the analysis was restricted to natural (*n* = 18; *R*^2^ = 0.117, *P* = 0.090) or non-natural (*n* = 14; *R*^2^ = 0.245, *P* = 0.042) menopause sub-groups.

## Discussion

In this study, our main finding is that premature/early menopause and nulliparity are associated with earlier onset of progressive multiple sclerosis. We also observed a ‘dose effect’ of number of pregnancies on delaying progressive multiple sclerosis. Although reproductive milestones of age at menarche and natural menopause did not differ between women with multiple sclerosis and population-based control women matched for year of birth, women with multiple sclerosis had lower number of pregnancies than control individuals. Developing progressive multiple sclerosis is the most important predictor of severe disability in multiple sclerosis (Paz Soldan *et al.*, 2015), and in our study, disability outcome expectedly mirrored the progressive multiple sclerosis disease course. Our study was able to at least partly address several important questions regarding reproductive period in women and multiple sclerosis. We discuss each of these questions in detail below.

### Does multiple sclerosis impact reproductive milestones in women?

Despite different methods of data collection between multiple sclerosis and control groups, distribution of reproductive milestones of menarche age and natural menopause age did not differ between the two groups. The mean age at natural menopause in our cohort was 50.2 ± 4.8 years, which was similar to the control women as well as the mean age of 51 for natural menopause in Western populations (Gold *et al.*, 2001; Harlow *et al.*, 2012) and in line with previous studies on multiple sclerosis (Bove *et al.*, 2016*b*; Baroncini *et al.*, 2019). This finding establishes that multiple sclerosis does not directly impact natural reproductive milestones in women (Bove *et al.*, 2016*b*).

However, we observed a higher number of multiple sclerosis patients who had non-natural menopause than controls. This finding may partially be due to mechanisms such as immunomodulation in addition to surgical causes leading to non-natural menopause in multiple sclerosis patients, whereas the main cause of non-natural menopause in control women is surgical menopause. Our study does not resolve this sub-question.

### Does menarche impact onset of relapsing-remitting or progressive phase of multiple sclerosis and/or attaining severe disability in multiple sclerosis?

Other studies in this area had previously suggested that earlier puberty is a risk factor for earlier onset of multiple sclerosis symptoms (Sloka *et al.*, 2006; Kavak *et al.*, 2015; Bove *et al.*, 2016*a*) and higher relapse rate (Lulu *et al.*, 2016). We found no association of age at menarche with age at onset of relapsing-remitting or progressive phase of multiple sclerosis or with attaining severe disability in multiple sclerosis in line with recent findings (Zuluaga *et al.*, 2019). However, our clinical series was specifically enriched for progressive multiple sclerosis; therefore, multiple sclerosis onset may be biased towards this specific population. Further prospective studies, particularly in paediatric multiple sclerosis, may better address this question, which remains inconclusively answered in our study.

### Do pregnancies impact onset of relapsing-remitting or progressive phase of multiple sclerosis and/or attaining severe disability in multiple sclerosis?

Our findings are in line with many of the previous studies that have shown that pregnancy even if terminated early can be protective of multiple sclerosis by reducing multiple sclerosis risk and delaying multiple sclerosis onset (Runmarker and Andersen, 1995; Holmqvist *et al.*, 2010; Kotzamani *et al.*, 2012; Magyari, 2015; Rejali *et al.*, 2016). Our finding of a dose effect of pregnancies on delaying the age at multiple sclerosis onset would be compatible with the previous findings of an association between a higher parity and a reduced risk of first attack (Ponsonby *et al.*, 2012). However, in the absence of age at pregnancy information, pregnancy could not be treated as a time-dependent variable; therefore, a definite conclusion about causality cannot be reached.

Relapse rate decreases during pregnancy (Confavreux *et al.*, 1998). In the postpartum period, there is an apparent early increase in number of relapses followed by a regression to the mean number of relapses to a similar rate as the year preceding the pregnancy (Vukusic *et al.*, 2004). This initial increase in disease activity after pregnancy (Houtchens *et al.*, 2018) could also lead to an earlier manifestation of symptoms of multiple sclerosis. This effect has been shown in the evolution of radiologically isolated syndrome to symptomatic multiple sclerosis (Lebrun *et al.*, 2012). Although, the previous findings are mostly on patients who used no disease-modifying therapies or discontinued first-line treatments before conception, in the modern treatment era, an increase in relapses during pregnancy has been reported, especially after discontinuation of natalizumab and fingolimod, which are linked to a higher rebound in disease activity (Alroughani *et al.*, 2018).

These findings are consistent with a greater degree of immune tolerance, mediated by high levels of oestradiol, in addition to other important hormones including progesterone and androgens (Airas *et al.*, 2008; Holmqvist *et al.*, 2010).

After multiple sclerosis onset, studies reported either no negative effect (Weinshenker *et al.*, 1989*b*; Confavreux *et al.*, 1998; Koch *et al.*, 2009; Ramagopalan *et al.*, 2012) or even a positive effect of pregnancy on accrual of disability (Verdru *et al.*, 1994; Runmarker and Andersen, 1995; D’Hooghe et al., 2012; Keyhanian *et al.*, 2012; Karp *et al.*, 2014). However, women who were diagnosed with multiple sclerosis and who had a more severe disease course tend to have fewer or no children (Runmarker and Andersen, 1995; Koch *et al.*, 2009). Similarly, in our study, multiple sclerosis patients had fewer pregnancies compared to the controls matched for the year of birth. Matching for the year or birth accounts for changing population behaviours in pregnancy rates over the years. Our finding is most likely related to either the intrinsic biology of multiple sclerosis or decisions by the patients due to having a chronic, potentially disabling disease.

We found a clear association with number of pregnancies and a delay in onset of progressive multiple sclerosis. Although most of the studies focussed on the impact of pregnancy on disability worsening in multiple sclerosis (Weinshenker *et al.*, 1989*b*; Verdru *et al.*, 1994; Runmarker and Andersen, 1995; Confavreux *et al.*, 1998; Koch *et al.*, 2009; D’Hooghe et al., 2012; Keyhanian *et al.*, 2012; Ramagopalan *et al.*, 2012; Karp *et al.*, 2014), only two prior studies investigated the impact of pregnancy on progressive multiple sclerosis onset. One study found a lower risk of developing SPMS in patients with higher parity (Runmarker and Andersen, 1995), whereas another study found no association (Koch *et al.*, 2009). Most patients develop progressive multiple sclerosis at an older age; therefore, it is more likely that pregnancies precede progressive multiple sclerosis onset than vice versa. Additionally, we observed an association between number of pregnancies and delayed onset of progressive multiple sclerosis, not only in the overall group but also after restricting the analysis to the group of postmenopausal women with progressive multiple sclerosis onset after menopause, thereby eliminating any chance that pregnancies happened after progressive multiple sclerosis onset.

Therefore, in our study, higher number of pregnancies likely delayed progressive multiple sclerosis onset and associated severe disability. We also found that, full-term or not, all pregnancies had a similar impact. Although we did not record the duration of incomplete pregnancies, our observation might suggest that any hormonal change associated with pregnancy, even when pregnancy was not successfully completed, has a delaying effect on progressive multiple sclerosis and multiple sclerosis-related disability. However, the sub-group analyses to help indirectly conclude on causality further suffer from loss of power due to lower number of patients. Future targeted studies may further clarify our findings regarding pregnancy dose and progressive multiple sclerosis outcome.

### Does menopause impact onset of relapsing-remitting or progressive phase of multiple sclerosis and/or attaining severe disability in multiple sclerosis?

Our study demonstrated an association between menopause and age at progressive multiple sclerosis onset or attaining severe disability even after restricting analyses to patients who had menopause before progressive multiple sclerosis onset. Expectedly, non-natural menopause, which was more common in multiple sclerosis patients was associated with premature menopause and was also associated with earlier age at onset of progressive multiple sclerosis. We also showed that premature or early menopause was associated with shorter time spent in relapsing-remitting phase and earlier onset of progressive multiple sclerosis.

Overall, regardless of mechanism, earlier menopause seems to have a detrimental association with long-term multiple sclerosis prognosis (Smith and Studd, 1992; Holmqvist *et al.*, 2006; Bove *et al.*, 2013, 2015, 2016*b*; Ladeira *et al.*, 2018; Baroncini *et al.*, 2019; Karageorgiou *et al.*, 2020). It remains unclear whether women who will develop progressive multiple sclerosis are more prone to early menopause or vice versa. However, distribution of age at natural menopause did not differ between patients with multiple sclerosis and matched controls in our study. It seems that multiple sclerosis or progressive disease (enriched in our study) does not impact menopause age. Therefore, we may suggest that our study confirms an impact of menopause on disease progression and not the vice versa.

There are several limitations to this study. We used patient-reported data, which may introduce recall bias, although we aimed to address this shortcoming by comparing to a population-based control group with a different method of data collection. The clinical series of multiple sclerosis patients from which the survey respondents were drawn was specifically enriched for progressive multiple sclerosis and, therefore, was also enriched for older patients and males with multiple sclerosis. A selection bias may still impact our interpretations due to differences between respondent and non-respondents to the survey. The patients who responded to the survey in our study were representative of the sex and age appropriate disease course distribution of a general multiple sclerosis population. However, because of the age of our study population, there was an expectedly higher number of older progressive multiple sclerosis patients with a higher motor disability level compared to younger relapsing-remitting multiple sclerosis patients. This dichotomy actually was an asset, because we seeked to study progressive multiple sclerosis outcome. However, the same dichotomy may have limited our conclusions regarding relapsing-remitting phase of multiple sclerosis. Additionally, we did not have detailed information on the potential impact of menopausal hormone therapies and our disability metric (EDSS) focussed mostly on motor function and did not capture significant cognitive disability. Finally, an independent impact of modern disease-modifying therapies on the outcome cannot be excluded, since the study group represented an older patient population who would have not received immunomodulation early in the disease course.

### Overall interpretation and the ‘hormone hypothesis in multiple sclerosis’

In general, despite all of our attempts to clarify directionality of associations, causality remains uncertain from association studies such as our study. However, the observed associations as discussed above, some unique to our study and some confirming other previous findings, between the number of pregnancies or menopause age, and progressive multiple sclerosis onset age also make biological sense. Although, pregnancy is a state of higher oestrogen levels, menopause represents a permanent cessation of ovarian follicular activity with a stable and persistent reduction of oestrogen levels (Baroncini *et al.*, 2019), but the biological process starts within 2–5 years of clinical menopausal transition (Hall, 2015). This gradual change in oestrogen levels fits the concept of an aging continuum being associated with a slow evolution, as opposed to a rapid phenotype switch, from relapsing-remitting to progressive disease in the fifth decade (Zeydan and Kantarci, 2018). There are also a number of observations about physiological changes associated with a reduction in oestrogen levels, and reported effects of oestrogen on neuroprotective processes pertinent to oligodendrocytes, neurons and microglia (Spence and Voskuhl, 2012; Villa *et al.*, 2016) as well as on anti-inflammatory processes, which also diminish with menopause (Gold and Voskuhl, 2009; Spence *et al.*, 2011; Christianson *et al.*, 2015). Our study supports the previously reported beneficial impact of oestrogen in multiple sclerosis, both by preventing relapses and delaying progressive multiple sclerosis onset.

While the type of oestrogen preparation does matter in terms of potential impact in multiple sclerosis, oestriol was shown to be immunomodulatory (Soldan *et al.*, 2003; Gold *et al.*, 2009) and to reduce number of enhancing lesions (Sicotte *et al.*, 2002). In a multicentre phase 2 trial, higher oestriol levels were associated with a reduction in relapses, cognitive improvement and dampening of cortical grey matter atrophy (Voskuhl *et al.*, 2016; MacKenzie-Graham *et al.*, 2018). Of course, such a neuroprotective effect cannot be suggested as being multiple sclerosis specific. However, assuming causality is proven in a follow-up study, higher oestriol levels during life due to multiple pregnancies and delayed menopause could have a potential beneficial effect on delaying progressive multiple sclerosis. Preclinical models of multiple sclerosis would further support this notion (Tiwari-Woodruff *et al.*, 2007; Crawford *et al.*, 2010; Ziehn *et al.*, 2012; Moore *et al.*, 2014; Kim *et al.*, 2018; Voskuhl *et al.*, 2019).

### Clinically relevant potential implications

Our study has potential implications on how women with multiple sclerosis can be counselled. For example, a pregnancy decision can potentially be reinforced in women with multiple sclerosis who may be doing well but had previously chosen to avoid pregnancy due to concerns about a negative impact of pregnancies in the future. Similarly, any decision about premature non-natural menopause should be discussed with the patient as a potentially harmful intervention. Possible use of menopausal hormone therapies should be reviewed with the patient. Ultimately, our study sets the stage for a prospective trial that is needed to prove if menopausal hormone therapies would effectively delay the onset of progressive multiple sclerosis in the setting of premature or early menopause. It should be noted that the type and dose of oestrogen preparation is also an important consideration for such a trial.

## Funding

This study was partly funded by National Institutes of Health (U54 AG044170). The population-based control females were derived from the Mayo Clinic Cohort Study of Oophorectomy and Aging-2 that was made possible by the Rochester Epidemiology Project (National Institutes of Health AG034676 and AG052425).

## Competing interests

B.Z., E.J.A., D.M.W., C.Y.S., W.A.R., L.G.R. and O.H.K. have no competing interests. B.M.K. has had research funded by Biogen Inc. and receives publishing royalties for Common Pitfalls in Multiple Sclerosis and CNS Demyelinating Diseases. B.G.W. receives royalties from RSR Ltd, Oxford University, Hospices Civil de Lyon and MVZ Labor PD Volkmann und Kollegen; serves as a member of an adjudication committee for clinical trials in NMO being conducted by MedImmune and Alexion; is a consultant for Caladrius Biosciences and Brainstorm Therapeutics; and serves as a member of a data safety monitoring committee for clinical trials conducted by Novartis. K.K. serves on data safety monitoring board for Takeda Global Research and Development Center, Inc. and receives research support from Avid Radiopharmaceuticals and Eli Lilly.

## Abbreviations

EDSS =: expanded disability status scale
IQR =: interquartile range
SD =: standard deviation
SPMS =: secondary progressive multiple sclerosis
